# Survival outcomes in endometrial cancer patients according to diabetes: a systematic review and meta-analysis

**DOI:** 10.1186/s12885-022-09510-7

**Published:** 2022-04-20

**Authors:** Lauren McVicker, Christopher R. Cardwell, Lauren Edge, W Glenn McCluggage, Declan Quinn, James Wylie, Úna C. McMenamin

**Affiliations:** 1 Centre for Public Health, Queen’s University Belfast, Belfast, Northern Ireland, UK; 2 Department of Pathology, Belfast Health and Social Care Trust, Grosvenor Road, Belfast, Northern Ireland, UK; 3 Department of Obstetrics and Gynaecology, Antrim Area Hospital, Northern Health and Social Care Trust, Antrim, Northern Ireland, UK

## Abstract

**Background:**

Diabetes is an established risk factor for endometrial cancer development but its impact on prognosis is unclear and epidemiological studies to date have produced inconsistent results. We aimed to conduct the first systematic review and meta-analysis to compare survival outcomes in endometrial cancer patients with and without pre-existing diabetes.

**Methods:**

We conducted a systematic search of MEDLINE, EMBASE and Web of Science databases up to February 2022 for observational studies that investigated the association between pre-existing diabetes and cancer-specific survival in endometrial cancer patients. Secondary outcomes included overall survival and progression or recurrence-free survival. Quality assessment of included studies was undertaken using the Newcastle–Ottawa Scale and a random-effects model was used to produce pooled hazard ratios (HRs) and 95% confidence intervals (CIs). (PROSPERO 2020 CRD42020196088).

**Results:**

In total, 31 studies were identified comprising 55,475 endometrial cancer patients. Pooled results suggested a worse cancer-specific survival in patients with compared to patients without diabetes (*n* = 17 studies, HR 1.15, 95% CI 1.00–1.32, I^2^ = 62%). Similar results were observed for progression or recurrence-free survival (*n* = 6 studies, HR 1.23, 95% CI 1.02–1.47, I^2^ = 0%) and for overall survival (*n* = 24 studies, HR 1.42, 95% CI 1.31–1.54, I^2^ = 46%).

**Conclusion:**

In this systematic review and meta-analysis, we show that diabetes is associated with a worse cancer-specific and overall survival in endometrial cancer patients.

**Supplementary Information:**

The online version contains supplementary material available at 10.1186/s12885-022-09510-7.

## Introduction

Endometrial cancer is the most common gynecological malignancy in Western countries and the sixth most common cancer among women globally and its incidence has increased markedly over the past two decades [1, 2]. In 2020, there were 544,000 new cases and 260,000 deaths from endometrial cancer globally, with the highest incidence and mortality rates in Northern America and Europe [1]. The more commonly occurring low-grade endometrioid carcinomas (previously referred to as type I carcinomas) are in general associated with a good prognosis [3]. In contrast, high-grade tumors (high-grade endometrioid and non-endometrioid), such as serous, clear cell carcinomas and undifferentiated carcinomas and carcinosarcomas (some of these previously referred to as type II carcinomas) are associated with a significantly worse prognosis with 5-year survival as low as 14% for some types [4–6].

There is evidence to suggest that many endometrial cancers (especially endometrioid-type) develop and progress in the context of metabolic dysfunction [7]. Obesity is an established risk factor for endometrial cancer [8] and is associated with a poorer overall survival [9]. Type 2 diabetes is also associated with endometrial cancer risk [10], with pooled analyses showing an up to two-fold increased risk, independent of body mass index (BMI) and physical activity levels [11, 12]. Insulin resistance and hyperinsulinemia are important features of diabetes and growing in vitro evidence suggests a direct effect of insulin and insulin-like growth factor 1 (IGF-1) on endometrial cancer [13, 14]. Increased cell proliferation and inhibition of apoptosis has been demonstrated with activation of the insulin receptor, most likely mediated through both the MAPK and PI3K/Akt pathways [14]. Despite mounting pre-clinical evidence, it is unclear how diabetes affects survival outcomes following a diagnosis of endometrial cancer and results from epidemiological studies have to date been conflicting.

Some studies have shown that women diagnosed with endometrial cancer who have diabetes are over twice as likely to die from their cancer compared to women without diabetes [15–18] while other studies have found no association [19, 20]. Racial disparities in endometrial cancer-specific survival according to diabetes status have also been noted in some [21], but not all studies [19]; these have suggested a poorer disease-specific survival in white endometrial cancer patients compared to black patients.

Two earlier systematic reviews (7 and 8 years ago) performed meta-analyses for the risk of death from endometrial cancer in women with compared to women without diabetes but only six studies were pooled and most followed cancer-free women until death from endometrial cancer [22, 23]. Therefore, death was used as a proxy for a cancer diagnosis making it difficult to disentangle the impact of diabetes on risk of endometrial cancer compared with survival from endometrial cancer [24]. Given the inconsistencies in epidemiological studies to date, we aimed to conduct the first systematic review and meta-analysis to determine the impact of pre-existing diabetes on cancer-specific survival in women diagnosed with endometrial cancer, and to investigate the risk of death according to important clinical and demographic factors.

## Methods

This systematic review and meta-analysis was conducted according to the Preferred Reporting Items for Systematic Reviews and Meta-analyses (PRISMA) guidelines [25]. Before commencement, the review was registered with the International prospective register of systematic reviews (PROSPERO 2020 CRD42020196088) [26].

### Search strategy

Three electronic databases were searched including MEDLINE (US National Library of Medicine, Bethesda, Maryland, USA), Embase (Reed Elsevier PLC, Amsterdam, Netherlands) and Web of Science (Thompson Reuters, Times Square, New York, USA) for relevant studies from database inception to 16^th^ February 2022. Keyword searches and Medical Subject Headings (MeSH) were used with no language restrictions. The search strategy used is listed in Additional file 1. The search was limited to humans and excluded reviews. Reference lists of the identified studies were also screened for eligible studies.

### Eligibility criteria

After removal of duplicates, all titles and abstracts were screened by at least two reviewers (LM, ÚM and LE). Full texts were independently screened by two reviewers and were included if they met the following criteria:

I. **Participants:** Women aged 18 or over who were diagnosed with endometrial cancer.
II. **Intervention/Exposure:** Diagnosis of diabetes mellitus (type 1 or type 2) before endometrial cancer, identified by self-report or through medical records.
III. **Comparators:** Endometrial cancer patients without a diabetes diagnosis.
IV. **Outcome(s):** Endometrial cancer-specific survival. Overall survival and progression or recurrence-free survival were secondary outcomes.

Studies were included if they reported a risk estimate and 95% confidence interval (CI) or if there was sufficient information provided to calculate an estimate. Abstracts without a full published text were included if they met the inclusion criteria and authors were contacted in an effort to gain more information, but none responded. Studies that determined diabetes status using only a single blood glucose measurement were not included as this is deemed to be insufficient for a clinical diagnosis of diabetes [27]. Additionally, if more than one study investigated survival outcomes in the same population, the study that investigated cancer-specific survival was included. Furthermore, if more than one study investigated cancer-specific survival within the same population, the largest study was prioritised for inclusion, or if they were of similar size, the study that considered the most confounders was included. Any discrepancies between authors as to whether a paper should be included was resolved through discussion.

### Data extraction and quality assessment

Data extraction was conducted independently by two reviewers and the following information was extracted from each study: author, year of publication, country, study design, study population, number of endometrial cancer patients, age of patients at diagnosis, average follow-up time, diabetes type, diabetes ascertainment method, outcomes investigated, number of outcomes, covariates adjusted for and study results. The Newcastle Ottawa Scale (NOS) was used to assess the quality of each of the studies [28].

## Statistical analysis

Statistical analysis was conducted using STATA version 16 software. Unadjusted and adjusted risk estimates (including odds ratio (OR), hazard ratio (HR) or relative risk (RR)) and corresponding 95% CI were extracted from each study. ORs (from one study [29]) and RRs (from two studies [18, 20]) were combined with HRs as ORs and RRs in this instance should roughly approximate a HR as endometrial cancer mortality is not a common outcome [30, 31]. One study [32] only presented a Kaplan Meier curve, from which the number of deaths were estimated and used to calculate a risk estimate and 95% CI using the indirect log hazard ratio and variance estimation method from Parmer et al. [33]. If studies presented results separately by endometrial cancer histology type (two studies [34, 35]), race (two studies [19, 21]) or follow-up time (one study [36]) these estimates were combined using a fixed effects model to produce one estimate before entering into the meta-analysis model[37]. As there was an overlap between the populations in two studies assessing endometrial cancer-specific survival, separate results were taken according to race; the Olson et al. [21] study was restricted to black endometrial cancer patients whilst the Lam et al. [38] study was restricted to white endometrial cancer patients, and treated separately. Additionally, one study [36] reported outcomes for endometrial cancer patients identified by two methods (cancer registry and National Health Service) and to avoid potential overlap in patients, only the risk estimate from patients identified from the cancer registry were included in the meta-analysis as this is deemed to be a higher quality source for cancer case identification [39].

As heterogeneity between individual studies was anticipated, a random effects model was used to combine a minimum of three studies reporting endometrial cancer-specific survival to produce an overall pooled estimate and 95% CI [40]. Adjusted estimates were prioritised for the meta-analysis but if not provided unadjusted estimates were used. Heterogeneity was assessed using the I-Squared statistic (*I*^*2*^); I^2^ values of 25%, 50% and 75% were considered low, moderate and high, respectively [41]. Secondary outcomes included overall survival and progression-free or recurrence-free survival. The definition of events for progression or recurrence-free survival varied between studies; one study only included disease progression [42], two only recurrence [43, 44], another either progression or recurrence [45] and two studies included recurrence or death [35, 46] as the end point.

Sub-group analyses were conducted by study quality; NOS score of ≤ 7 or NOS score of > 7, study type (population-based or institution-based), average follow-up duration (< 5 years or ≥ 5 years) and diabetes ascertainment method (self-reported or medical records/diabetes register). If there was a minimum of two studies, sub-group analysis was also undertaken by histological tumor type (endometrioid or non-endometrioid) and race (black or white). Where possible, sub-group analysis was undertaken restricted to studies that adjusted for or stratified by BMI. Lastly, sub-group analysis was performed for studies where all or at least 90% of endometrial cancer patients had undergone surgery.

Sensitivity analyses were performed restricting to studies with adjusted results only and restricting to studies that reported a HR for endometrial cancer-specific survival. An additional analysis systematically removing each study in order to determine its effect on the main pooled estimate was conducted. To check for publication bias, funnel plots were produced and visually inspected for asymmetry and additionally Egger’s test of funnel plot asymmetry was applied [47]. The Trim and Fill method was used to attempt to calculate a pooled estimate whilst adjusting for any funnel plot asymmetry [48].

## Results

### Study selection

**Fig. 1:**
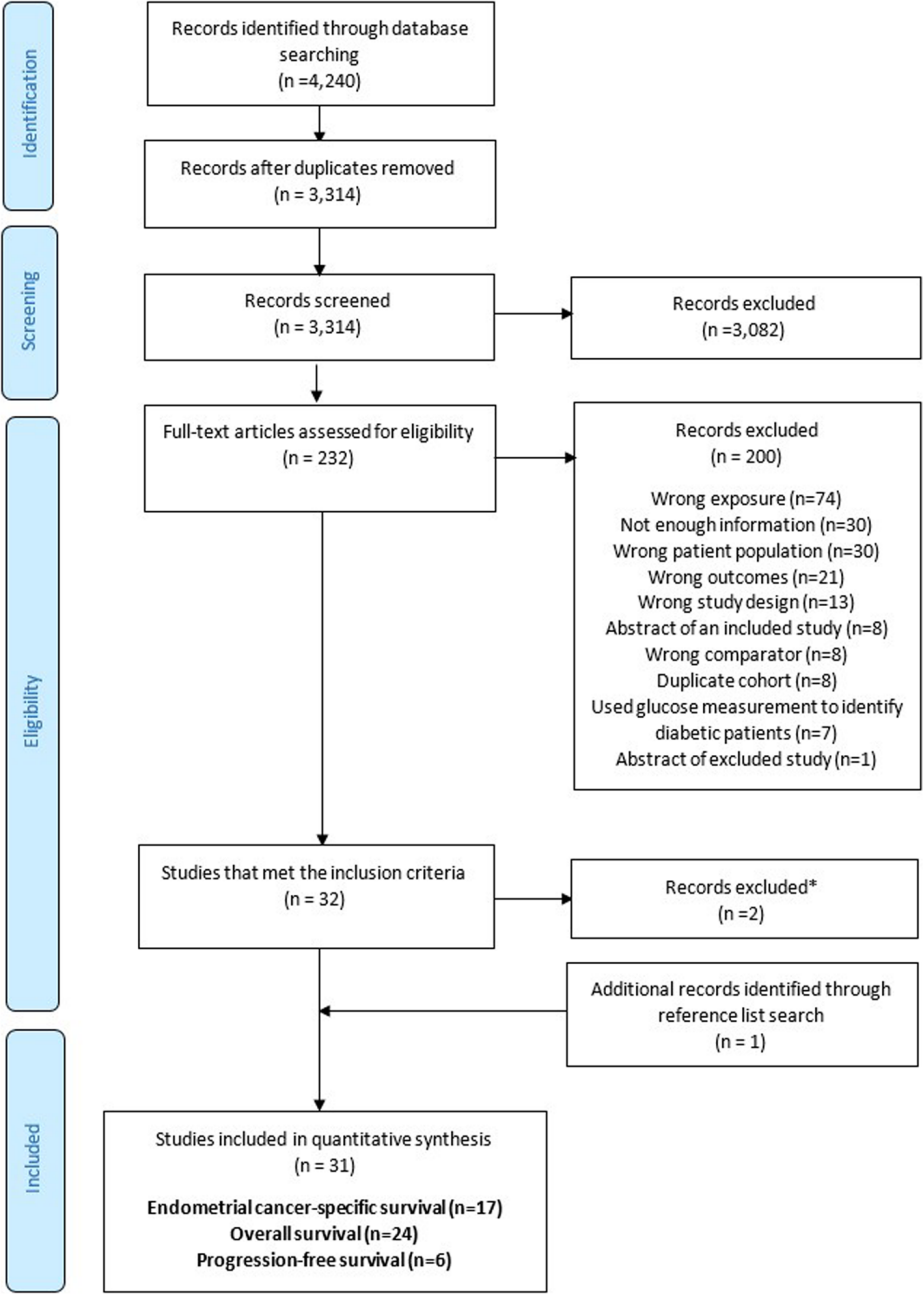
Flow diagram of the study selection process. *One study and one abstract were excluded as they reported an inconsistent risk estimate and 95% confidence interval

After removal of duplicates, 3,314 records were screened by title and abstract. A total of 232 articles were identified for full text review and of these, 32 studies met the inclusion criteria (see Fig. 1). Two studies were subsequently excluded as the risk estimate was not consistent with the 95% CI reported [49, 50]. An additional study [34] was identified by searching the reference lists of the included studies. This resulted in a total of 31 studies (30 full text articles and 1 abstract [51]), 17 of which reported endometrial cancer-specific survival, 24 overall survival and 6 progression-free or recurrence-free survival. Ten studies reported both endometrial cancer-specific survival and overall survival.

### Study characteristics

The characteristics of the studies that investigated endometrial cancer-specific survival and overall survival are outlined in Tables 1 and 2, respectively. There were 15 studies conducted in the U.S. [15, 18, 19, 21, 32, 34, 35, 38, 42, 46, 51–55], nine in European countries, including Sweden [56], Norway [17], the United Kingdom (U.K) [57], the Netherlands [58], Finland [59], Poland [43, 60, 61], Latvia [36], Germany [45] and France [29]. One study was conducted in Australia [16], one in Taiwan [62], one in Brazil [44] and two studies combined records from institutions in different countries; one from institutions in the U.S. and China [20] and the other from institutions in Germany and Japan [63]. All studies, except one [55], were cohorts in design; 20 were based in single or multicentre institutions and 11 were population-based. The mean or median age of endometrial cancer patients at study entry was 60 years or older in most studies. Eleven studies only included endometrial cancer patients who had undergone surgery [29, 32, 34, 42–44, 46, 51, 52, 59, 60], and three studies reported that at least 90% of patients had undergone surgery [16, 18, 61]. Nine studies specifically included patients with type 2 diabetes [18, 32, 52, 56, 59–62] or “adult onset diabetes” [53] and the remaining studies did not specify diabetes type for inclusion. The most common method of diabetes ascertainment was through medical records (*n* = 17), while other methods included self-report (*n* = 8) and a diabetes register (*n* = 2). In all studies, diabetes status was ascertained at or before endometrial cancer diagnosis.

**Table 1:** Characteristics of studies reporting cancer-specific survival for endometrial cancer patients with diabetes compared to without

Author, year and location	Study design	Study cohort source/database	Endometrial cancer study population	Recruitment period	Study size	Diabetes ascertainment	No. of EC-specific deaths	Risk estimate reported	Outcome ascertainment	Follow-up (mean/ median)	NOS score
Bjornsdottir[56] 2020 Sweden	Population-based	Swedish Cancer Registry	NR	1998–2012	NR	The Swedish National Diabetes Register	NR	HR	The Total Population Register and Swedish Cause of Death Registry	6.6 years (median)	7
Brandt[51] 2019 U.S	Single institution-based	Memorial Sloan Kettering Cancer Centre	Stage I	2006–2016	1458	Medical records	NR	HR	NR	2.4 years (median)	6
Donkers [57] 2021 U.K	Single institution-based	Royal Cornwall Hospital Trust Truro, Cornwall	High grade EC stages I-IV	2006–2017	176	Medical records	70	HR	Medical records	NR	8
Felix[34] 2015 U.S	Multi institution-based	NRG Oncology/Gynecologic Oncology Group 210 molecular staging trial	Stages I-IV	2003–2011	4109	Self-reported	582	HR	Medical records and cancer registry	5 years (median)	6
Folsom[18] 2004 U.S	Population-based	Iowa women’s health study and SEER cancer registry	Stages I-IV	1986–2000	415	Self-reported	39	RR	Registry, questionnaires and NDI	6.4 years (median)	6
Kolehmainen [59] 2020 Finland	Single institution-based	Obstetrics and Gynaecology, Helsinki University Hospital	Stages I-IV	2007–2012	515	Self-reported	97	HR	Medical records and death certificates	6.75 years (median)	4
Lam[38] 2018 U.S	Population-based	SEER database	Stages I-IV	2000–2011	22,743 ^a b^	Medicare claims	NR	HR	SEER database	NR	8
Lees [53] 2021 U.S	Population-based	Wisconsin Cancer Registry	Stages I-IV	2006–2016	745	Structured interview	46	HR	National death index	19.9 years (median)	8
Lindemann[17] 2015 Norway	Population-based	Health surveys of North Trondelag (HUNT)	Stages I-IV	NR	337	Self-reported	56	HR	Cancer registry	6.7 years (median)	5
Nagle[16] 2018 Australia	Population-based	Australian National EC Study	Stages I-IV	2005–2007	1359	Self-reported	123	HR	Medical records and NDI	7.1 years (median)	6
Olson[21] 2012 U.S	Population-based	SEER-Medicare database	Stages I-IV	2000- 2005	958 ^c^	Medicare claims	NR	HR	SEER registries	3.5 years (median)	6
Ribeiro [44] 2021 Brazil	Single institution-based	Department of Obstetrics and Gynaecology, University of Campinas, Sao Paulo	Non-endometrioid EC stages I-IV	2002–2017	127	Medical records	NR	HR	Medical records	4.5 years (mean)	5
Ruterbusch[19] 2014 U.S	Single institution-based	Henry Ford Health System (HFHS) tumor registry	Stages I-IV	1990–2005	627	HFHS, medical records and the MDCSS	109	HR	HFHS, medical records and the MDCSS	NR	6
Simon [54] 2021 U.S	Population-based	Women’s Health Initiative (WHI)	Stages I-III	1993–1998	NR	Self-report and medication use	NR	HR	Medical records, death certificates, relative’s report and NDI	10 years (median)	6
Sung[20] 2000 U.S. and China	Multi-institution-based	Women & Infants’ Hospital of RI, Cornell University Medical Centre, Shanghai Medical University, and Women’s and Children’s Hospital, University of Southern California	Stages I-IV	1976 to 1998	125	Medical records	112	RR	Medical records and pathology reports	5.4 years (mean)	7
VanArsdale[15] 2019 U.S	Single institution-based	Montefiore Medical Centre	Stages I-IV	1999–2016	1,732	Medical records	393	HR	Social security death index	3.4 years (median)	7
Zanders[58] 2013 Netherlands	Population-based	Eindhoven cancer registry	Endometrioid EC stages I-III	2000- 2008	388	Medical records	NR	HR	Municipal personal records	NR	8

^a^Included all uterine cancer patients

^b^Only white patients included due to cohort overlap with Olson et al.[21]

^c^Only black patients included due to cohort overlap with Lam et al.[38]

*EC* Endometrial cancer, *NOS* Newcastle Ottawa Scale, *NDI* National Death Index, *SEER* Surveillance, Epidemiology and End results program, *MDCSS* Metropolitan Detroit Cancer Surveillance System, *NR* Not reported, *U.S.* United States, *RI* Rhode Island, *HR* Hazard Ratio, *RR* Relative Risk

**Table 2:** Characteristics of studies reporting overall survival for endometrial cancer patients with diabetes compared to without

Author, year and location	Study Design	Study cohort source/ database	Endometrial cancer study population	Recruitment period	Study size	Diabetes ascertainment	No. of overall deaths	Risk estimate reported	Outcome ascertainment	Follow-up (mean/ median)	NOS score
AlHilli[42] 2016 U.S	Single institution-based	Mayo Clinic	Stage I-IV	1999–2008	516 ^a^	NR	NR	HR	Medical records, tumor registry and death certificates	4.3 years for DM, 5.2 years for non-DM (median)	7
Bjornsdottir [56] 2020 Sweden	Population-based	Swedish Cancer Registry	NR	1998–2012	NR	The Swedish National Diabetes Register	NR	HR	The Total Population Register and Swedish Cause of Death Registry	6.6 years (median)	7
Chen[62] 2016 Taiwan	Single institution-based	Chang Gung Memorial hospital	Stage I-IV	2000–2010	1450 ^b^	Hospital admissions	89	HR	NR	NR	5
Donkers [57] 2021 U.K	Single institution-based	Royal Cornwall Hospital Trust Truro, Cornwall	High grade EC stages I-IV	2006–2017	176	Medical records	96	HR	Medical records	NR	8
Folsom[18] 2004 U.S	Population-based	Iowa women’s health study and SEER cancer registry	Stages I-IV	1986–2000	415	Self-reported	93	RR	Registry, questionnaires and NDI	6.4 years (median)	6
Gottwald[60] 2011 Poland	Single institution-based	N. Copernicus Memorial Provincial Specialist Hospital	Endometrioid EC stages I-III	2000–2007	260	NR	49	HR	Medical records	NR	3
Hein[45] 2020 Germany	Single institution-based	University of Erlangen	Stages I-IV	1987–2010	287	Patient files or questionnaire	113	HR	Clinical cancer registry	NR	5
Ko[35] 2014 U.S	Multi-institution-based	University of North Carolina hospital and Duke Cancer Institute	Stages I-IV	2005- 2010	1411	Medical records	NR	HR	NR	NR	4
Kolehmainen [59] 2020 Finland	Single institution-based	Obstetrics and Gynaecology, Helsinki University Hospital	Stages I-IV	2007–2012	515	Self-reported	160	HR	Medical records and death certificates	6.75 years (median)	4
Kusne [55] 2020 U.S	Single institution-based	Mayo Clinic Hospital	Stages I-IV	2006–2016	96 ^a^	Medical records	6	HR	Medical records	3.9 years (median)	5
Larouzée[29] 2019 France	Population-based	Echantillon Généraliste de Bénéficiaires (EGB)	Stage NR	2005–2014	405	EGB database	155	OR	EGB database	NR	6
Lees [53] 2021 U.S	Population-based	Wisconsin Cancer Registry	Stages I-IV	2006–2016	745	Structured interview	450	HR	National death index	19.9 years (median)	8
Lemánska[61] 2015 Poland	Single institution-based	Poznan University of Medical Sciences	Stages I-III	2002–2010	107	Medical database	NR	HR	Greater Poland cancer registry	NR	5
Liang[46] 2016 U.S	Single institution -based	Memorial Sloan Kettering Cancer Centre	High grade stage IA	1995- 2012	85	NR	6	HR	Medical records	3.9 years (median)	5
Lindemann[17] 2015 Norway	Population-based	Health surveys of North Trondelag (HUNT)	Stages I-IV	NR	337	Self-reported	166	HR	Cancer registry	6.7 years (median)	5
Linder[43] 2006 Poland	Single institution -based	Maria Sklodowska-Curie Memorial Cancer Centre	Stage I-III	1992- 1998	880	NR	NR	HR	NR	8.8 years (median)	6
Nagle[16] 2018 Australia	Population-based	Australian National EC Study	Stages I-IV	2005–2007	1359	Self-reported	179	HR	Medical records and NDI	7.1 years (median)	6
Nicholas[52] 2014 U.S	Single institution -based	Tertiary care centre	Stages I-IV	1992–2008	490	Medical records and self-report	NR	HR	Physician records, hospital cancer registry and obituary surveillance	4.5 years (median)	5
Olson[21] 2012 U.S	Population-based	SEER-Medicare database	Stages I-IV	2000- 2005	12,568	Medicare claims	5,123	HR	SEER registries	3.5 years (median)	6
Ruterbusch[19] 2014 U.S	Single institution-based	Henry Ford Health System (HFHS) tumor registry	Stages I-IV	1990–2005	627	HFHS, medical records and the MDCSS	320	HR	HFHS, medical records and the MDCSS	NR	6
Steiner[63] 2007 Germany and Japan	Multi-institution-based	University hospital and the University of Hokkaido	Stages I-IV	1985–2003	297	Hospital records	75	HR	NR	3.7 years (mean)	4
Stevens[32] 2012 U.S	Multi-institution-based	Medical centres at the State University of New York and Tumor registry	Endometrioid EC stages I-IV	2000–2010	82	Medical records and tumor registry	25	HR	Tumor registry, medical records and the SSI	NR	5
Strele[36] 2018 Latvia	Population-based cohort	Centre for Disease Prevention and Control of Latvia (cancer registry)	Stages I-IV	2009–2013	1,685	Diabetes register	470	HR	Causes of Death database	2.5 years for DM and 2.7 years for non-DM (median)	6
Zanders[58] 2013 Netherlands	Population-based	Eindhoven cancer registry	Endometrioid EC stages I-III	2000- 2008	1644	Medical records	310	HR	Municipal personal records	NR	8

^a^Restricted to matched/propensity score matched EC patients

^b^Included all uterine cancer patients

*EC* Endometrial cancer, *NOS* Newcastle Ottawa Scale, DM Diabetes Mellitus, NDI National Death Index, *SEER* Surveillance, Epidemiology and End results program. *MDCSS* Metropolitan Detroit Cancer Surveillance System, *SSI* Social Security Index, *NR* Not reported, *U.S.* United States, *HR* Hazard Ratio, *OR* Odds Ratio, *RR* Relative Risk

The factors adjusted for in each of the studies is summarised in Additional file 2. Most of the studies adjusted for a number of potential confounders while three studies reported unadjusted estimates [32, 46, 61] and one study reported only an age-adjusted estimate [62]. Cancer stage was adjusted for in 15 of 17 studies that assessed cancer-specific survival, and 14 of 24 studies that assessed overall survival. Seven studies [20, 38, 42, 43, 54, 57, 58] adjusted for comorbidities such as cardiovascular disease, hypertension and cerebrovascular disease. The majority of studies had an NOS score of 5 or above, Tables 1 and 2.

### Endometrial cancer-specific survival

**Fig. 2:**
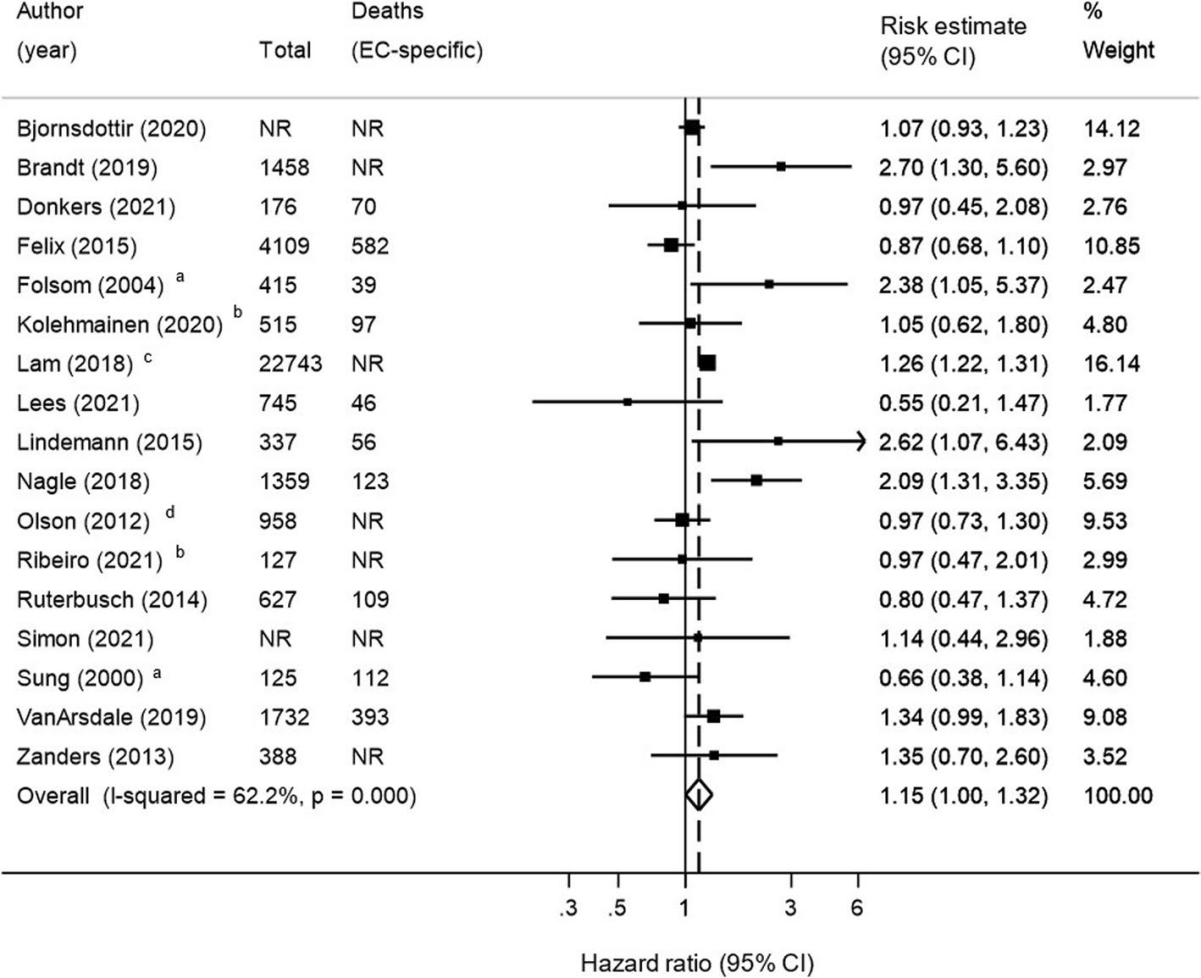
Forest plot showing the Hazard Ratio and 95% CI for cancer-specific survival in endometrial cancer patients with diabetes compared to without. ^a^Relative risk. ^b^Unadjusted estimate. ^c^Only white patients included due to cohort overlap [21]. ^d^Only black patients included due to cohort overlap [38]. EC = Endometrial cancer. CI = Confidence Interval. NR = Not Reported

Seventeen studies investigated endometrial cancer-specific survival, and in the meta-analysis comprising 35,814 patients, those with diabetes had a significant 15% increased risk of cancer death compared to patients without diabetes (HR 1.15, 95% CI 1.00–1.32), with moderate heterogeneity observed (I^2^ = 62%; *P* < 0.01), see Fig. 2.

Sub-group analyses are presented in Table 3. Results were similar according to study quality but were statistically significant when restricted to higher quality studies (HR 1.23, 95% CI 1.07–1.42, I^2^ = 6.4%). The risk of cancer-specific mortality for patients with diabetes was more marked when restricting to population-based studies (HR 1.24, 95% CI 1.05–1.47, I^2^ = 61%) compared to institution-based studies and heterogeneity increased (HR 1.04, 95% CI 0.79–1.37, I^2^ = 59%). Results were similar when stratified by length of follow-up, Table 3. In sub-group analysis by diabetes ascertainment method, risk of cancer-specific death was more marked in studies where diabetes was self-reported, albeit not significantly (HR 1.32, 95% CI 0.87–1.99, I^2^ = 70%). Six studies adjusted for BMI and when pooled, no significant difference was seen for patients with diabetes compared to those without diabetes (HR 0.94, 95% CI 0.72–1.23, I^2^ = 41%). When restricting to four studies of patients diagnosed with endometrioid endometrial carcinoma, results were similar to the main analysis although not statistically significant (HR 1.20, 95% CI 0.84–1.72, I^2^ = 42%) and results were further attenuated for non-endometrioid endometrial carcinoma types, Table 3. In sub-group analysis of six studies restricting to endometrial cancer patients who had undergone surgery, risk of death from endometrial cancer was higher for patients with diabetes compared to patients without, although it did not reach statistical significance (HR 1.44, 95% CI 0.93–2.22, I^2^ = 75%). In stratified analysis by race, diabetes was associated with a significant increased risk of death from endometrial cancer in white patients (HR 1.26, 95% CI 1.22–1.31, I^2^ = 0%), whereas no association was observed in black patients (HR 0.92, 95% CI 0.71–1.20, I^2^=0%).

**Table 3:** Analysis results for cancer-specific and overall survival in endometrial cancer patients with compared to without diabetes

	**No. of included studies**	**No. of EC patients**	**Pooled estimate (95% CI)**	**I-Squared (%)**	**P**_**heterogeneity**_** value**
**Endometrial cancer-specific survival**					
Main analysis	17	35,814	1.15 (1.00–1.32)	62.2	< 0.01
Multivariate analysis	15	35,172	1.17 (1.00–1.35)	66.3	< 0.01
Restricting to studies that reported a HR	15	35,274	1.16 (1.07–1.33)	59.8	< 0.01
Studies with a quality score of ≤ 7	13	11,762	1.17 (0.97–1.42)	61.2	< 0.01
Studies with a quality score of > 7	4	24,052	1.23 (1.07–1.42)	6.4	0.36
Studies with a follow-up of < 5 years ^a^	4	4,275	1.27 (0.89–1.82)	60.7	0.05
Studies with a follow-up of ≥ 5 years ^a^	9	7,605	1.15 (0.88–1.49)	65.1	< 0.01
Population-based studies	9	26,945	1.24 (1.05–1.47)	61.0	< 0.01
Institution-based studies	7	8,742	1.04 (0.79–1.37)	59.6	0.02
Self-reported diabetes	7	7,480	1.32 (0.87–1.99)	69.7	< 0.01
Medical record reported diabetes	10	28,334	1.13 (0.98–1.30)	57.5	0.01
Endometrioid histology	4	4,4453	1.20 (0.84–1.72)	41.6	0.16
Non-endometrioid histology	4	2,079	1.00 (0.81–1.23)	0.0	0.47
Restricted to white women	2	23,099	1.26 (1.22–1.31)	0.0	0.62
Restricted to black women	2	1,229	0.92 (0.71–1.20)	0.0	0.40
Adjusted for BMI ^b^	6	6,887	0.94 (0.72–1.23)	40.6	0.01
Undergone surgery	6	7,983	1.44 (0.93–2.22)	75.0	< 0.01
**Overall survival**					
Main analysis	24	26,352	1.42 (1.31–1.54)	46.3	0.01
Multivariate analysis	20	25,563	1.43 (1.31–1.56)	54.3	< 0.01
Univariate analysis	9	5,998	1.33 (1.21–1.46)	0.0	0.79
Studies with a quality score of ≤ 7	21	23,787	1.41 (1.29–1.54)	47.1	0.01
Studies with a quality score of > 7	3	2,565	1.45 (1.12–1.88)	38.4	0.20
Studies with a follow-up of < 5 years ^a^	6	15,136	1.28 (1.21–1.37)	0.0	0.48
Studies with a follow-up of ≥ 5 years ^a^	8	4,767	1.65 (1.32–2.06)	76.8	< 0.01
Population-based studies	9	19,158	1.43 (1.27–1.61)	72.9	< 0.01
Institution-based studies	15	7,194	1.43 (1.28–1.61)	0.0	0.70
Self-reported diabetes	5	3,371	1.99 (1.61–2.46)	32.8	0.20
Medical record reported diabetes	15	21,240	1.28 (1.22–1.34)	0.0	0.92
Endometrioid histology	5	3,196	1.63 (1.18–2.25)	41.2	0.15
Restricted to white women	2	11,966	1.35 (1.09–1.65)	41.9	0.19
Restricted to black women	2	1,229	1.27 (1.08–1.50)	0.0	0.63
Adjusted for BMI	7	5,374	1.52 (1.22–1.90)	59.6	0.02
Overweight and obese patients (≥ 25 kg/m^2^ or ≥ 30 kg/m^2^)	2	824	1.76 (1.19–2.61)	0.0	0.93
Undergone surgery	11	5,209	1.57 (1.28–1.93)	62.7	< 0.01

^a^Only included studies which reported a mean or median follow-up

^b^Sung et al. adjusted for obesity

*EC* Endometrial cancer, *CI* Confidence Interval, *HR* Hazard Ratio

In sensitivity analyses, excluding two studies that reported RRs (see Table 3) or the exclusion of individual studies (data not shown) did not markedly change the pooled cancer-specific mortality risk estimate. There was no evidence of publication bias in the funnel plot for studies reporting endometrial cancer-specific survival (see Additional file 3).

### Overall survival

**Fig. 3:**
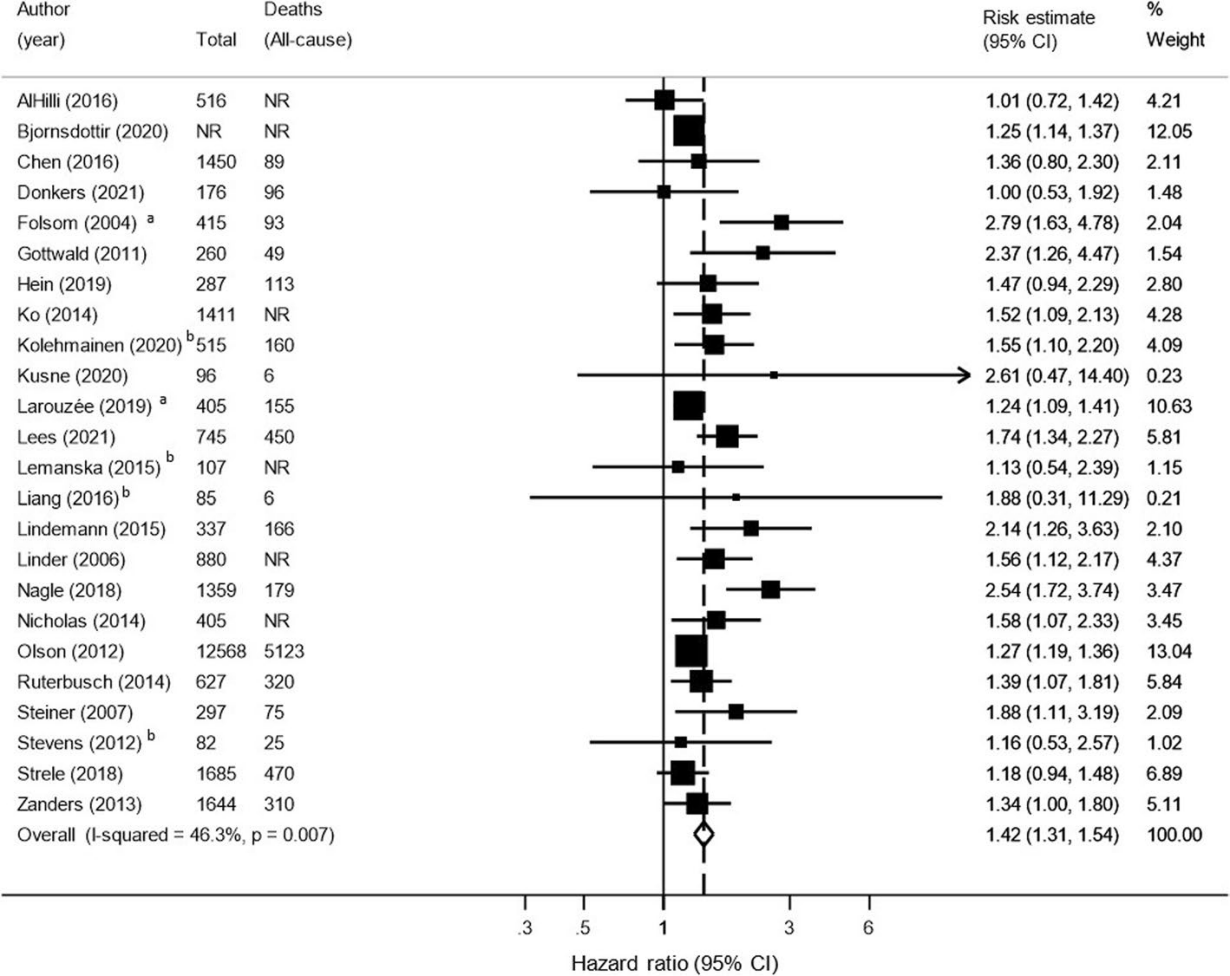
Forest plot showing the Hazard Ratio and 95% CI for overall survival in endometrial cancer patients with diabetes compared to without. ^a^ Odds ratio or relative risk. ^b^ Unadjusted estimate. CI = Confidence Interval. NR = Not Reported

Twenty-four studies investigated overall survival in 26,352 endometrial cancer patients and the pooled HR for patients with diabetes compared to patients without diabetes was 1.42 (95% CI 1.31–1.54) with moderate heterogeneity observed (I^2^ = 46%, *P* = 0.01), as shown in Fig. 3.

There was a consistently higher risk of death from any cause observed for patients with diabetes compared to patients without diabetes across all sub-groups, see Table 3. A more marked increase in risk was observed when restricting to patients with endometrioid cancer and in patients who were overweight or obese, (endometrioid: HR 1.63, 95% CI 1.18–2.25, I^2^ = 41%, overweight or obese: HR 1.76, 95% CI 1.19–2.61, I^2^ = 0%, respectively). White patients with diabetes had a slightly higher risk of death from any cause when compared to black patients with diabetes (white patients: HR 1.35, 95% CI 1.09–1.65, black patients: 1.27, 95% CI 1.08–1.50). In addition, when restricting to studies that adjusted for BMI, patients with diabetes had a significantly higher risk of death from any cause (HR 1.52, 95% CI 1.22–1.90, I^2^ = 60%).

In sensitivity analysis, the exclusion of individual studies did not markedly change the pooled overall survival estimate (data not shown). Upon visual inspection of the funnel plot for studies reporting overall survival there appeared to be asymmetry (see Additional file 4), which may reflect publication bias (Egger’s test *P* < 0.01). Using the Trim and Fill method, imputation of eight potentially missing studies to adjust for asymmetry resulted in an attenuated, albeit still increased risk of death from any cause for patients with diabetes (*n* = 32 studies, HR 1.30, 95% CI 1.18–1.42).

### Progression or recurrence-free survival

Endometrial cancer patients with diabetes had a significant 23% increased risk of disease progression or recurrence compared to patients without diabetes (*n* = 6 studies, HR 1.23, 95% CI 1.02–1.47, I^2^ = 0%, *P* = 0.88) and results were similar across sub-group analyses, see Additional files 5 and 6.

## Discussion

In this systematic review and meta-analysis we found that endometrial cancer patients with pre-existing diabetes had a significant 15% increased risk of cancer-specific death compared to patients without diabetes but this was not consistent across all sub-group analyses. Patients with diabetes experienced a significant 42% and 23% higher risk of all-cause mortality and progression or recurrence of disease, respectively compared to patients without diabetes which was generally similar across sub-group analyses.

Mechanisms relating to hyperglycaemia and hyperinsulinemia may underlie the observed increased risk of cancer-specific death in endometrial cancer patients with diabetes. Pre-clinical evidence has shown that endometrial tumor cells have altered glucose metabolism compared to normal endometrial cells and this can facilitate proliferation, adhesion and invasion [64]. Up to 93% of so-called Type I endometrial tumors (endometrioid type) are missing the phosphatase and tensin homologue (PTEN), or have mutations in the PI3K/Akt/mTOR pathways that it regulates [64, 65]. These pathways support glucose uptake (glucose transporters) and metabolism in order to fuel cell growth [64, 66, 67]. In endometrial cancer patients with diabetes, high levels of blood glucose may directly promote proliferation of tumor cells by providing the required glucose-derived carbon for these pathways [64, 68]. In patients without hyperinsulinemia, IGF binding proteins (IGFBP-1 and IGFBP-2) control the amount of active IGF-1 that can act on cells to induce cell proliferation [13]. However, in patients with hyperinsulinemia not only is more insulin secreted, but the production of IGFBP-1 and IGFBP-2 is diminished, therefore permitting a higher concentration of active IGF-1 which can stimulate insulin receptors (IR) on endometrial tumor cells [13, 14]. Stimulation of the IRs and IGF-IRs has been shown to upregulate proliferation of endometrial tumor cells and inhibit apoptosis through the MAPK and PI3K/Akt/mTOR pathways [13, 14]. Furthermore, high expression of IGF-1 receptor has been linked to adverse prognostic factors such as lymph node involvement, even after controlling for age, BMI and histological type; it is therefore plausible that hyperinsulinemia may have a direct influence on the progression of endometrial cancer [69]. Similar to cancer-specific mortality, we found that progression or recurrence-free survival was significantly poorer in endometrial cancer patients with diabetes compared to those without diabetes, even when restricting to studies that reported multivariate estimates (including adjustment for BMI). Only six studies however, were identified in this secondary analysis and studies differed in their definitions of tumor progression that limited interpretation and exploration of sub-groups.

Obesity is a common risk factor for both diabetes and endometrial cancer and has been associated with poorer overall survival [9] among endometrial cancer patients; however, its influence on endometrial cancer-specific mortality is less clear [70]. Due to a lack of studies, we were unable to conduct stratified analysis for cancer-specific survival according to BMI categories but in a sensitivity analysis restricting to studies that adjusted for BMI [15, 20, 34, 53, 54, 57], there was no difference in survival according to diabetes status. Higher amounts of adipose tissue has been shown to increase the conversion of androgens to estrogens, which when unopposed by progesterone in the endometrium can result in increased endometrial proliferation, hyperplasia and cancer development [70]. The lack of association between diabetes and cancer-specific survival among studies that controlled for BMI may suggest that the association between diabetes and cancer-specific survival is mediated by BMI, but only six studies adjusted for this in their analyses and further investigation is required. Moreover, definitions of BMI varied between studies and one study [20] only adjusted for obesity. Additionally, patients who have morbid obesity and associated comorbidities that contraindicate surgery may be treated more conservatively [71], increasing the likelihood of disease progression. When restricting to patients who underwent surgery there was still evidence of an increased risk of cancer-specific death among patients with diabetes but this was not statistically significant, possibly reflecting the reduced number of studies.

None of the identified studies in this review included sufficient information on diabetes severity or duration. Although diabetes is a risk factor for endometrial cancer, the influence of diabetes duration on endometrial cancer risk is also poorly understood. In a recent investigation in the Nurses’ Health Study and Health Professionals Follow-up Study [10], the risk of endometrial cancer, amongst other cancer types, reached highest levels at 4–8 years after type 2 diabetes diagnosis after which the elevated risk did not further increase, therefore aligning with the gradual diminishment of endogenous production of insulin over time. The authors concluded that this finding, together with analysis of clinical measures for endogenous insulin secretion and long-term blood glucose concentration over time (C-peptide and HbA1c, respectively), support the hypothesis that insulin resistance and hyperinsulinemia has a direct impact on cancer pathogenesis and development, rather than representing a spurious association confounded by shared risk factors between type 2 diabetes and cancer [10]. Similar investigations in longitudinal endometrial cancer cohorts are required to evaluate the influence of diabetes duration, as well as diabetes type and severity, on long-term cancer outcomes.

In sub-group analysis by histological tumor type, there was a suggestion that diabetes was associated with a higher risk of endometrial cancer-specific death in endometrioid in comparison to non-endometrioid cancers, albeit this was not statistically significant and only four studies were included. In sub-group analyses by race, we found that the increase in cancer-specific death was seen only in white endometrial cancer patients and no significant association was observed among black patients with diabetes, despite adjusting for tumor stage, grade and histology. These findings by race may be reflective of the type of endometrial cancer; in the US, endometrioid carcinoma rates are highest in non-Hispanic whites whereas non-endometrioid carcinoma rates are highest in non-Hispanic blacks [72], so it is possible that diabetes is less likely to influence the progression of non-endometrioid tumors. Only two studies, however, stratified results by race and numbers were substantially reduced in the black sub–group so these novel associations warrant further investigation in large population-based cohorts.

Overall survival was a secondary outcome in this review and the meta-analysis of 24 studies showed that endometrial cancer patients with pre-existing diabetes had a 42% higher risk of death from any cause compared to patients without diabetes, which was generally consistent across all sub-group analyses. Diabetes is a major contributor to morbidity and mortality through microvascular (neuropathy and nephropathy) and macrovascular (cardiovascular disease (CVD), stroke and peripheral vascular disease) complications [73]. Of particular concern, CVD accounts for 65% of all deaths in people with diabetes, and is the leading cause of death for endometrial cancer patients [73, 74]. CVD therefore likely contributed to the higher risk of all-cause mortality demonstrated in our meta-analysis. Only four studies [42, 43, 57, 58] investigating overall survival adjusted their analysis for comorbidities (including CVD), two of which still observed an increased risk of death among endometrial cancer patients with pre-existing diabetes compared to patients without diabetes [43, 58]. Risk of all-cause death was notably higher for patients with diabetes even when restricted to studies that adjusted for BMI, which has previously been suggested to be an independent risk factor for all-cause mortality in endometrial cancer patients [70].

This is the first systematic review and meta-analysis to our knowledge to investigate the risk of cancer-specific mortality in endometrial cancer patients according to diabetes. The review has a number of strengths, including the use of a comprehensive search strategy in three large databases with no language restrictions. Moreover, all articles were independently screened and reference lists were reviewed to ensure all relevant studies were identified. Eleven of the included studies were population-based and although study sizes varied, pooled analysis of cancer-specific mortality included over 35,800 endometrial cancer patients. Although the number of studies was reduced, we conducted novel meta-analyses for sub-groups based on tumor histology and race.

Some potential limitations of our review should also be acknowledged. Although most studies adjusted for age at diagnosis, cancer stage, type and grade, other factors varied and likely explained some of the moderate heterogeneity observed in pooled analyses. As discussed, there is potential for residual confounding by factors such as comorbidities, lifestyle factors and race/ethnicity since not all studies adjusted for these. Some studies also lacked information on cancer treatments and there is some evidence that cancer patients with diabetes experience less aggressive cancer treatment compared to patients without diabetes, which may contribute to poorer survival [75, 76], however, an increased risk of endometrial cancer-specific mortality was still observed in individual studies that controlled for cancer treatments [15, 38, 58] and suggested in pooled sub-group analyses restricted to patients who underwent surgery. Most studies included patients with any type of diabetes, but given the much lower prevalence of type 1 diabetes [77], as well as the average age of endometrial cancer onset (60 years) [78], it can likely be assumed that the majority of diabetes cases were type 2. Eight studies were based on self-reported diabetes, but validation studies have shown good correlation with clinical diagnoses of diabetes across a number of different settings [79–81]. Four studies received a quality score of less than five, and inclusion of low quality primary studies has been shown to exaggerate the overall estimate produced in meta-analysis [82], however in sub-group analysis restricting to studies with a high quality score, pooled results were similar to the main analysis. We used the NOS tool for quality assessment and although widely used, we acknowledge that this tool may not have assessed all aspects of observational studies and other tools are available [83]. Additionally, asymmetry was noted in the funnel plot for studies investigating overall survival which could reflect publication bias. Reassuringly, using the trim and fill method [48] to approximate the pooled estimate after imputation of eight potentially missing studies the association between diabetes and all-cause mortality was only partly attenuated. Lastly, the pooling of OR, RRs and HRs together in a meta-analysis is debated, however, only three of 31 studies reported a RR or OR and as endometrial cancer-specific mortality is not a common outcome, OR and RRs should largely approximate a HR in this instance [31]. Furthermore, in sensitivity analysis excluding studies that did not report HRs, the pooled estimate was unchanged. Despite these limitations, the findings from this systematic review could inform future prognostic models for endometrial cancer to supplement established clinical and histopathological factors [3], with the ultimate aim of better identifying endometrial cancer patient groups at highest risk of progression, in whom risk-reducing treatments can be better targeted. The recently proposed molecular classification of endometrial cancer [84] (identified in The Cancer Genome Atlas [66]) will offer future opportunities for further prognostic stratification of endometrial cancer.

## Conclusion

In the first systematic review and meta-analysis to evaluate the association between diabetes and endometrial cancer progression, there was suggestive evidence that diabetes was associated with a worse cancer-specific survival, but there was insufficient data for detailed sub-group analysis by important clinical and demographic factors. To establish whether diabetes is an important prognostic feature in endometrial cancer, further investigation is required in large population-based studies with detailed information on diabetes type, duration and severity as well as patient and tumor factors to enable the conduct of sub-group analyses. The findings of our review and meta-analysis may be useful in counselling patients with diabetes who develop endometrial cancer.

## Supplementary Information

**Additional file 1: Table S1.** Database searchterms.

**Additional file 2: Table S2.** Factors adjusted for in each of the included studies.

**Additional file 3: Figure S1.** Funnel plot for cancer-specific survival studies.

**Additional file 4: Figure S2.** Funnel plot for overall survival studies.

**Additional file 5: Figure S3.** Forest plot for progression or recurrence-free survival in endometrial cancer patients with diabetes compared to without.

**Additional file 6: Table S3.** Analyses for progression or recurrence-free survival in endometrial cancer patients with diabetes compared to without.

## Data Availability

Not applicable.

## References

[1] Sung H, Ferlay J, Siegel RL, Laversanne M, Soerjomataram I, Jemal A (2021). Global cancer statistics 2020: GLOBOCAN estimates of incidence and mortality worldwide for 36 cancers in 185 countries. CA Cancer J Clin.

[2] Sheikh MA, Althouse AD, Freese KE, Soisson S, Edwards RP, Welburn S (2014). USA endometrial cancer projections to 2030: should we be concerned?. Future Oncol.

[3] Morice P, Leary A, Creutzberg C, Abu-Rustum N, Darai E (2016). Endometrial cancer. The Lancet.

[4] Bendifallah S, Ouldamer L, Lavoue V, Canlorbe G, Raimond E, Coutant C (2017). Patterns of recurrence and outcomes in surgically treated women with endometrial cancer according to ESMO-ESGO-ESTRO Consensus Conference risk groups: Results from the FRANCOGYN study Group. Gynecol Oncol.

[5] Colombo N, Preti E, Landoni F, Carinelli S, Colombo A, Marini C (2013). Endometrial cancer: ESMO Clinical Practice Guidelines for diagnosis, treatment and follow-up. Ann Oncol.

[6] Nama N, Cason FD, Misra S, Hai S, Tucci V, Haq F (2020). Carcinosarcoma of the Uterus: A Study From the Surveillance Epidemiology and End Result (SEER) Database. Cureus.

[7] Bjørge T, Stocks T, Lukanova A, Tretli S, Selmer R, Manjer J (2010). Metabolic Syndrome and Endometrial Carcinoma. Am J Epidemiol.

[8] Aune D, Navarro Rosenblatt DA, Chan DS, Vingeliene S, Abar L, Vieira AR (2015). Anthropometric factors and endometrial cancer risk: a systematic review and dose-response meta-analysis of prospective studies. Ann Oncol.

[9] Secord AA, Hasselblad V, Von Gruenigen VE, Gehrig PA, Modesitt SC, Bae-Jump V (2016). Body mass index and mortality in endometrial cancer: A systematic review and meta-analysis. Gynecol Oncol.

[10] Hu Y, Zhang X, Ma Y, Yuan C, Wang M, Wu K (2020). Incident Type 2 Diabetes Duration and Cancer Risk: A Prospective Study in Two US Cohorts. J Natl Cancer Inst.

[11] Friberg E, Orsini N, Mantzoros CS, Wolk A (2007). Diabetes mellitus and risk of endometrial cancer: a meta-analysis. Diabetologia.

[12] Sciacca L, Vigneri R, Tumminia A, Frasca F, Squatrito S, Frittitta L (2013). Clinical and molecular mechanisms favoring cancer initiation and progression in diabetic patients. Nutr Metab Cardiovasc Dis.

[13] Renehan AG, Frystyk J, Flyvbjerg A (2006). Obesity and cancer risk: the role of the insulin-IGF axis. Trends Endocrinol Metab.

[14] Nagamani M, Stuart CA (1998). Specific binding and growth-promoting activity of insulin in endometrial cancer cells in culture. Am J Obstet Gynecol.

[15] Van Arsdale A, Miller DT, Kuo DY, Isani S, Sanchez L, Nevadunsky NS (2019). Association of obesity with survival in patients with endometrial cancer. Gynecol Oncol.

[16] Nagle CM, Crosbie EJ, Brand A, Obermair A, Oehler MK, Quinn M (2018). The association between diabetes, comorbidities, body mass index and all-cause and cause-specific mortality among women with endometrial cancer. Gynecol Oncol.

[17] Lindemann K, Cvancarova M, Eskild A (2015). Body mass index, diabetes and survival after diagnosis of endometrial cancer: A report from the HUNT-Survey. Gynecol Oncol.

[18] Folsom AR, Anderson KE, Sweeney C, Jacobs DRJ (2004). Diabetes as a risk factor for death following endometrial cancer. Gynecol Oncol.

[19] Ruterbusch JJ, Ali-Fehmi R, Olson SH, Sealy-Jefferson S, Rybicki BA, Hensley-Alford S (2014). The influence of comorbid conditions on racial disparities in endometrial cancer survival. Am J Obstet Gynecol.

[20] Sung CJ, Zheng Y, Quddus MR, Kang X, Zhang ZF, Lauchlan SC (2000). p53 as a significant prognostic marker in endometrial carcinoma. Int J Gynecol Cancer.

[21] Olson SH, Atoria CL, Cote ML, Cook LS, Rastogi R, Soslow RA (2012). The impact of race and comorbidity on survival in endometrial cancer. Cancer Epidemiol Biomarkers Prev.

[22] Liao C, Zhang D, Mungo C, Tompkins DA, Zeidan AM (2014). Is diabetes mellitus associated with increased incidence and disease-specific mortality in endometrial cancer? A systematic review and meta-analysis of cohort studies. Gynecol Oncol.

[23] Zhang ZH, Su PY, Hao JH, Sun YH (2013). The role of preexisting diabetes mellitus on incidence and mortality of endometrial cancer: a meta-analysis of prospective cohort studies. Int J Gynecol Cancer.

[24] Renehan AG, Yeh HC, Johnson JA, Wild SH, Gale EAM, Møller H (2012). Diabetes and cancer (2): evaluating the impact of diabetes on mortality in patients with cancer. Diabetologia.

[25] Page MJ, McKenzie JE, Bossuyt PM, Boutron I, Hoffmann TC, Mulrow CD (2021). The PRISMA 2020 statement: an updated guideline for reporting systematic reviews. BMJ.

[26] McVicker L, Edge L, Cardwell C, McMenamin Ú. The impact of diabetes on survival in endometrial cancer patients: a systematic review and meta-analysis: PROSPERO 2020 CRD42020196088 2020 [18–08–2020]. Available from: https://www.crd.york.ac.uk/prospero/display_record.php?RecordID=196088.

[27] Diagnostic Criteria For Diabetes: Diabetes UK; [13–11–2020]. Available from: https://www.diabetes.org.uk/professionals/position-statements-reports/diagnosis-ongoing-management-monitoring/new_diagnostic_criteria_for_diabetes.

[28] Wells G, Shea S, D OC. The Newcastle-Ottawa Scale (NOS) for assessing the quality of Nonrandomised studies in meta-analyses. 2011 [10–08–19]. Available from: http://www.ohri.ca/programs/clinical_epidemiology/oxford.asp.

[29] Larouzée E, Phelippeau J, Roberti E, Koskas M (2019). Evaluation of the French medical practices in endometrial cancer management by using quality indicators. Eur J Obstet Gynecol Reprod Biol.

[30] Ranganathan P, Aggarwal R, Pramesh CS (2015). Common pitfalls in statistical analysis: Odds versus risk. Perspect Clin Res.

[31] Symons MJ, Moore DT (2002). Hazard rate ratio and prospective epidemiological studies. J Clin Epidemiol.

[32] Stevens EE, Yu S, Van Sise M, Pradhan TS, Lee V, Pearl ML (2012). Hemoglobin A1c and the relationship to stage and grade of endometrial cancer. Arch Gynecol Obstet.

[33] Parmar MK, Torri V, Stewart L (1998). Extracting summary statistics to perform meta-analyses of the published literature for survival endpoints. Stat Med.

[34] Felix AS, Scott McMeekin D, Mutch D, Walker JL, Creasman WT, Cohn DE (2015). Associations between etiologic factors and mortality after endometrial cancer diagnosis: the NRG Oncology/Gynecologic Oncology Group 210 trial. Gynecol Oncol.

[35] Ko EM, Walter P, Clark L, Jackson A, Franasiak J, Bolac C (2014). The complex triad of obesity, diabetes and race in Type I and II endometrial cancers: prevalence and prognostic significance. Gynecol Oncol.

[36] Strele I, Pildava S, Repsa I, Kojalo U, Vilmanis J, Brigis G (2018). Pre-existing diabetes mellitus and all-cause mortality in cancer patients: a register-based study in Latvia. Acta Oncol.

[37] Barili F, Parolari A, Kappetein PA, Freemantle N (2018). Statistical Primer: heterogeneity, random- or fixed-effects model analyses?. Interact Cardiovasc Thorac Surg.

[38] Lam C, Cronin K, Ballard R, Mariotto A (2018). Differences in cancer survival among white and black cancer patients by presence of diabetes mellitus: Estimations based on SEER-Medicare-linked data resource. Cancer Med.

[39] Yarnell J, O'Reilly D, editors. Epidemiology and disease prevention. A global approach. 2nd ed. United Kingdom: Oxford University Press; 2013. p. 263–4.

[40] DerSimonian R, Laird N. Meta-analysis in clinical trials. Control Clin Trials. 1986;7(3):177–88. 10.1016/0197-2456(86)90046-2 3802833

[41] Higgins JPT, Thompson SG, Deeks JJ (2003). Measuring inconsistency in meta-analyses. BMJ.

[42] Al Hilli MM, Bakkum-Gamez JN, Mariani A, Cliby WA, Mc Gree ME, Weaver AL (2016). The effect of diabetes and metformin on clinical outcomes is negligible in risk-adjusted endometrial cancer cohorts. Gynecol Oncol.

[43] Linder B, Krynicki R, Michalski W, Jonska-Gmyrek J (2006). Endometrial cancer and concomitant diseases. Gin Onkol.

[44] Ribeiro CM, Brito LGO, Benetti-Pinto CL, Teixeira JC, Yela DA (2021). Is Diagnostic Hysteroscopy Safe for the Investigation of Type II Endometrial Cancer? A Retrospective Cohort Analysis. J Minim Invasive Gynecol.

[45] Hein A, Schneider MO, Renner SK, Fasching PA, Fiessler C, Titz S (2020). Risk of postmenopausal hormone therapy and patient history factors for the survival rate in women with endometrial carcinoma. Arch Gynecol Obstet.

[46] Liang LW, Perez AR, Cangemi NA, Zhou Q, Iasonos A, Abu-Rustum N (2016). An Assessment of Prognostic Factors, Adjuvant Treatment, and Outcomes of Stage IA Polyp-Limited Versus Endometrium-Limited Type II Endometrial Carcinoma. Int J Gynecol Cancer.

[47] Egger M, Smith GD, Schneider M, Minder C (1997). Bias in meta-analysis detected by a simple, graphical test. BMJ.

[48] Duval S, Tweedie R (2000). Trim and fill: A simple funnel-plot-based method of testing and adjusting for publication bias in meta-analysis. Biometrics.

[49] Kucukoztas N, Dizdar O, Rahatli S, Tarhan C, Haberal N, Dursun P (2013). Impact of comorbidities on recurrence rates and survival in patients with endometrial cancer. J Clin Oncol.

[50] Deng Y, Wang J, Li W, Xiong W, Wang X (2020). Efficacy of metformin in the treatment of estrogen-dependent endometrial carcinoma complicated with type 2 diabetes mellitus and analysis of its prognosis. Journal of BUON : official journal of the Balkan Union of Oncology.

[51] Brandt B, Basaran D, Shepherd C, Ali N, Thompson E, Leong K (2019). The prognostic impact of clinical and uterine factors in stage I endometrial cancer with negative lymph-vascular space invasion. Int J Gynecol Cancer.

[52] Nicholas Z, Hu N, Ying J, Soisson P, Dodson M, Gaffney DK (2014). Impact of comorbid conditions on survival in endometrial cancer. Am J Clin Oncol.

[53] Lees B, Hampton JM, Trentham-Dietz A, Newcomb P, Spencer R (2021). A population-based study of causes of death after endometrial cancer according to major risk factors. Gynecol Oncol.

[54] Simon MS, Hastert TA, Barac A, Banack HR, Caan BJ, Chlebowski RT (2021). Cardiometabolic risk factors and survival after cancer in the Women's Health Initiative. Cancer.

[55] Kusne YN, Kosiorek HE, Buras MR, Coppola KE, Verona PM, Cook CB (2020). Mortality and glycemic control among patients with diabetes mellitus and uterine or ovarian cancer. Future Sci OA.

[56] Bjornsdottir HH, Rawshani A, Rawshani A, Franzen S, Svensson A-M, Sattar N (2020). A national observation study of cancer incidence and mortality risks in type 2 diabetes compared to the background population over time. Sci Rep.

[57] Donkers H, Fasmer KE, McGrane J, Pijnenborg JMA, Bekkers R, Haldorsen IS (2021). Obesity and visceral fat: Survival impact in high-grade endometrial cancer. Eur J Obstet Gynecol Reprod Biol.

[58] Zanders MM, Boll D, van Steenbergen LN, van de Poll-Franse LV, Haak HR (2013). Effect of diabetes on endometrial cancer recurrence and survival. Maturitas.

[59] Kolehmainen A, Pasanen A, Tuomi T, Koivisto-Korander R, Bützow R, Loukovaara M (2020). Clinical factors as prognostic variables among molecular subgroups of endometrial cancer. PLOS ONE.

[60] Gottwald L, Chałubińska J, Moszyńska-Zielińska M, Piekarski J, Tyliński W, Szwalski J (2011). Endometrioid endometrial cancer–the prognostic value of selected clinical and pathological parameters. Ginekol Pol.

[61] Lemańska A, Zaborowski M, Spaczyński M, Nowak-Markwitz E (2015). Do endometrial cancer patients benefit from metformin intake?. Ginekol Pol.

[62] Chen JY, Chiou WK, Chou WY, Lin JD (2016). The impact of type 2 diabetes mellitus on mortality in hospitalized female cancer patients in Taiwan. Asia Pac J Clin Oncol.

[63] Steiner E, Plata K, Interthal C, Schmidt M, Faldum A, Hengstler JG (2007). Diabetes mellitus is a multivariate independent prognostic factor in endometrial carcinoma: a clinicopathologic study on 313 patients. Eur J Gynaecol Oncol.

[64] Byrne FL, Martin AR, Kosasih M, Caruana BT, Farrell R (2020). The Role of Hyperglycemia in Endometrial Cancer Pathogenesis. Cancers.

[65] Hecht J, Mutter G (2006). Molecular and pathologic aspects of endometrial carcinogenesis. J Clin Oncol.

[66] Levine DA, Network CGAR (2013). Integrated genomic characterization of endometrial carcinoma. Nature.

[67] Memarzadeh S, Zong Y, Janzen DM, Goldstein AS, Cheng D, Kurita T (2010). Cell-autonomous activation of the PI3-kinase pathway initiates endometrial cancer from adult uterine epithelium. Proc Natl Acad Sci.

[68] Byrne FL, Poon IK, Modesitt SC, Tomsig JL, Chow JD, Healy ME (2014). Metabolic vulnerabilities in endometrial cancer. Cancer Res.

[69] Joehlin-Price AS, Stephens JA, Zhang J, Backes FJ, Cohn DE, Suarez AA (2016). Endometrial Cancer Insulin-Like Growth Factor 1 Receptor (IGF1R) Expression Increases with Body Mass Index and Is Associated with Pathologic Extent and Prognosis. Cancer Epidemiol Biomarkers Prev.

[70] Arem H, Irwin ML (2013). Obesity and endometrial cancer survival: a systematic review. Int J Obes (Lond).

[71] Papadia A, Ragni N, Salom EM (2006). The impact of obesity on surgery in gynecological oncology: a review. International Journal of Gynecologic Cancer.

[72] Clarke MA, Devesa SS, Harvey SV, Wentzensen N (2019). Hysterectomy-Corrected Uterine Corpus Cancer Incidence Trends and Differences in Relative Survival Reveal Racial Disparities and Rising Rates of Nonendometrioid Cancers. J Clin Oncol.

[73] Deshpande AD, Harris-Hayes M, Schootman M (2008). Epidemiology of Diabetes and Diabetes-Related Complications. Physl Ther.

[74] Ward KK, Shah NR, Saenz CC, McHale MT, Alvarez EA, Plaxe SC (2012). Cardiovascular disease is the leading cause of death among endometrial cancer patients. Gynecol Oncol.

[75] van de Poll-Franse LV, Houterman S, Janssen-Heijnen ML, Dercksen MW, Coebergh JW, Haak HR (2007). Less aggressive treatment and worse overall survival in cancer patients with diabetes: a large population based analysis. Int J Cancer.

[76] Truong PT, Kader HA, Lacy B, Lesperance M, MacNeil MV, Berthelet E (2005). The effects of age and comorbidity on treatment and outcomes in women with endometrial cancer. Am J Clin Oncol.

[77] Xu G, Liu B, Sun Y, Du Y, Snetselaar LG, Hu FB (2018). Prevalence of diagnosed type 1 and type 2 diabetes among US adults in 2016 and 2017: population based study. BMJ..

[78] Key Statistics for Endometrial Cancer: American Cancer Society; 2021. Available from: https://www.cancer.org/cancer/endometrial-cancer/about/key-statistics.html#:~:text=Endometrial%20cancer%20affects%20mainly%20post,likely%20to%20die%20from%20it.

[79] Pastorino S, Richards M, Hardy R, Abington J, Wills A, Kuh D (2015). Validation of self-reported diagnosis of diabetes in the 1946 British birth cohort. Prim Care Diabetes.

[80] Oksanen T, Kivimäki M, Pentti J, Virtanen M, Klaukka T, Vahtera J (2010). Self-Report as an Indicator of Incident Disease. Ann Epidemiol.

[81] Comino EJ, Tran DT, Haas M, Flack J, Jalaludin B, Jorm L (2013). Validating self-report of diabetes use by participants in the 45 and up study: a record linkage study. BMC Health Serv Res.

[82] Khan KS, Daya S, Jadad A (1996). The importance of quality of primary studies in producing unbiased systematic reviews. Arch Intern Med.

[83] Higgins JPT, Thomas J, Chandler J, Cumpston M, Li T, Page MJ, Welch VA, editors. Cochrane handbook for systematic reviews of interventions. 2nd ed. Chichester: Wiley; 2019.

[84] Talhouk A, McConechy MK, Leung S, Li-Chang HH, Kwon JS, Melnyk N (2015). A clinically applicable molecular-based classification for endometrial cancers. Br J Cancer.

