# Supplementary material for: Survival outcomes in endometrial cancer patients according to diabetes: a systematic review and meta-analysis
Source: BMC Cancer. 2022 Apr 20;22:427. doi: 10.1186/s12885-022-09510-7 (PMC9019948; doi:10.1186/s12885-022-09510-7)
Supplement: Supplementary file 1 — Additional file 1: Table S1. Database searchterms. [file 12885_2022_9510_MOESM1_ESM.docx]

| **Table S1**. Search terms |
| --- |
| (cancer* OR neoplas* OR neoplasms/ OR tumo?r* OR carcinoma* OR Carcinoma/ OR adenocarcinoma* OR Adenocarcinoma/ OR malignan* OR adenosarcoma* OR Adenosarcoma/ OR “clear cell carcinoma*” OR carcinosarcoma* OR Carcinosarcoma/) |
| AND |
| (endometr* OR Endometrium/ OR Myometrium/ OR myometr* OR uter* OR Uterus/ OR womb) |
| AND |
| (Diabetes Mellitus/ OR diabet* OR “type 2 diabet*” OR Diabetes Mellitus, Type 2/ OR “type II diabet*” OR “T2D*” OR “type two diabet*” OR “insulin dependent diabet*” OR “IDDM*” OR “non-insulin dependent diabet*” OR “NIDDM” OR Hyperglycemia/ OR hypergly?emi* OR “insulin resistan*” OR Insulin Resistance/ OR Hyperinsulinism/ OR hyperinsulin* OR Metabolic Syndrome/ OR “metabol* syndrome*” OR “dm1” OR “dm2”) |
| AND |
| (Death/ OR mortalit* OR Mortality/ OR survival OR Survival/ OR Disease Progression/ OR “disease progression” OR prognosis OR Prognosis/ OR relapse OR recurrence OR Recurrence/ OR “overall survival” OR “cancer-specific survival” OR “progression-free survival” OR “disease-free survival” OR Disease-Free Survival/ OR Progression-Free Survival/ OR “recurrence-free survival” OR Follow-Up Studies/ OR “follow-up” OR Survival Analysis/ OR “survival analysis” OR Survival Rate/ OR “ survival rate”). |
