# Supplementary material for: Survival outcomes in endometrial cancer patients according to diabetes: a systematic review and meta-analysis
Source: BMC Cancer. 2022 Apr 20;22:427. doi: 10.1186/s12885-022-09510-7 (PMC9019948; doi:10.1186/s12885-022-09510-7)
Supplement: Supplementary file 2 — Additional file 2: Table S2. Factors adjusted for in each of the included studies. [file 12885_2022_9510_MOESM2_ESM.docx]

| **Table S2**. **Factors adjusted for in each of the included studies**. | | | | | | | | | | | | | | | | | | |
| --- | --- | --- | --- | --- | --- | --- | --- | --- | --- | --- | --- | --- | --- | --- | --- | --- | --- | --- |
| **Author (year)** | **Age** | **Year of diagnosis** | **Race** | **Cancer stage** | **Cancer grade** | **Cancer histology** | **Cancer treatment** | **Body mass index** | **Comorbidities ^a^** | **Medications ^b^** | **Smoking status/history** | **Reproductive factors ^c^** | **Family history** | **Prior cancer** | **Performance status ^d^** | **Education** | **Other ^e^** |  |
| *Cancer-specific survival* | | | | | | | | | | | | | | | | | |  |
| Bjornsdottir (2020) | **✓** |  |  |  |  |  |  |  |  |  |  |  |  |  |  | **✓** | **✓** |  |
| Brandt (2019) | **✓** |  |  | **✓** | **✓** |  |  |  |  |  |  |  |  |  |  |  |  |  |
| Donkers (2020) | **✓** |  |  | **✓** | **✓** | **✓** |  | **✓** | **✓** |  |  |  |  |  |  |  | **✓** |  |
| Felix (2015) | **✓** |  | **✓** | **✓** |  |  |  | **✓** |  | **✓** | **✓** | **✓** |  | **✓** |  | **✓** | **✓** |  |
| Folsom (2004) | **✓** |  |  | **✓** | **✓** |  |  |  |  |  |  |  |  |  |  |  |  |  |
| Kolehmainen (2020) |  |  |  |  |  |  |  |  |  |  |  |  |  |  |  |  |  |  |
| Lam (2018) | **✓** |  | **✓** | **✓** |  |  | **✓** |  | **✓** |  |  |  |  |  |  |  |  |  |
| Lees (2021) | **✓** |  |  | **✓** |  |  |  | **✓** |  |  | **✓** |  |  |  |  |  | **✓** |  |
| Lindemann (2015) | **✓** |  |  | **✓** |  | **✓** |  |  |  |  |  |  |  |  |  |  |  |  |
| Nagle (2018) | **✓** |  |  | **✓** | **✓** | **✓** |  |  |  |  |  |  |  |  |  |  |  |  |
| Olson (2012) | **✓** | **✓** | **✓** | **✓** | **✓** | **✓** | **✓** |  |  |  |  |  |  | **✓** |  | **✓** | **✓** |  |
| Ribeiro (2021) |  |  |  |  |  |  |  |  |  |  |  |  |  |  |  |  |  |  |
| Ruterbusch (2014) | **✓** | **✓** |  | **✓** | **✓** | **✓** | **✓** |  |  |  |  |  |  |  |  |  | **✓** |  |
| Simon (2021) | **✓** |  | **✓** |  |  |  |  | **✓** | **✓** | **✓** | **✓** |  |  |  |  | **✓** | **✓** |  |
| Sung (2000) ^f^ | **✓** |  | **✓** | **✓** | **✓** |  |  | **✓** | **✓** | **✓** |  |  |  | **✓** |  |  | **✓** |  |
| VanArsdale (2019) | **✓** |  | **✓** | **✓** | **✓** | **✓** | **✓** | **✓** |  | **✓** |  |  |  |  |  |  |  |  |
| Zanders (2013) | **✓** | **✓** |  | **✓** |  |  | **✓** |  | **✓** | **✓** |  |  |  |  |  |  |  |  |
| *Overall survival* | | | | | | | | | | | | | | | | | |  |
| AlHilli (2016) | **✓** |  |  | **✓** | **✓** | **✓** | **✓** | **✓** | **✓** |  | **✓** |  |  |  | **✓** |  | **✓** |  |
| Bjornsdottir (2020) | **✓** |  |  |  |  |  |  |  |  |  |  |  |  |  |  | **✓** | **✓** |  |
| Chen (2016) | **✓** |  |  |  |  |  |  |  |  |  |  |  |  |  |  |  |  |  |
| Donkers (2020) | **✓** |  |  | **✓** | **✓** | **✓** |  | **✓** | **✓** |  |  |  |  |  |  |  | **✓** |  |
| Folsom (2004) | **✓** |  |  | **✓** | **✓** |  |  |  |  |  |  |  |  |  |  |  |  |  |
| Gottwald (2011) | **✓** |  |  | **✓** | **✓** | **✓** |  |  |  |  |  | **✓** | **✓** |  |  |  | **✓** |  |
| Hein (2020) | **✓** |  |  | **✓** |  | **✓** |  | **✓** |  | **✓** |  |  |  |  |  |  |  |  |
| Ko (2014) | **✓** |  | **✓** | **✓** | **✓** | **✓** | **✓** | **✓** |  |  |  |  |  |  |  |  |  |  |
| Kolehmainen |  |  |  |  |  |  |  |  |  |  |  |  |  |  |  |  |  |  |
| Kusne (2020) | **✓** |  |  |  |  |  |  |  |  |  |  |  |  |  |  |  |  |  |
| Larouzée (2019) | **✓** |  |  |  |  |  |  |  |  |  |  |  |  |  |  |  | **✓** |  |
| Lees (2021) | **✓** |  |  | **✓** |  |  |  | **✓** |  |  | **✓** |  |  |  |  |  | **✓** |  |
| Lemanska (2015) |  |  |  |  |  |  |  |  |  |  |  |  |  |  |  |  |  |  |
| Liang (2016) |  |  |  |  |  |  |  |  |  |  |  |  |  |  |  |  |  |  |
| Lindemann (2015) | **✓** |  |  | **✓** |  | **✓** |  |  |  |  |  |  |  |  |  |  |  |  |

| **Table S2 Continued.** | | | | | | | | | | | | | | | | | |
| --- | --- | --- | --- | --- | --- | --- | --- | --- | --- | --- | --- | --- | --- | --- | --- | --- | --- |
| **Author (year)** | **Age** | **Year of diagnosis** | **Race** | **Cancer stage** | **Cancer grade** | **Cancer histology** | **Cancer treatment** | **Body mass index** | **Comorbidities ^a^** | **Medications ^b^** | **Smoking status/history** | **Reproductive factors ^c^** | **Family history** | **Prior cancer** | **Performance status ^d^** | **Education** | **Other ^e^** |
| Linder (2006) | **✓** |  |  | **✓** | **✓** | **✓** |  | **✓** | **✓** |  |  |  |  |  | **✓** |  | **✓** |
| Nagle (2018) | **✓** |  |  | **✓** | **✓** | **✓** |  | **✓** |  |  |  |  |  |  |  |  |  |
| Nicholas (2014) | **✓** |  |  | **✓** | **✓** |  |  |  |  |  |  |  |  |  |  |  |  |
| Olson (2012) | **✓** | **✓** |  | **✓** | **✓** | **✓** | **✓** |  |  |  |  |  |  | **✓** |  | **✓** | **✓** |
| Ruterbusch (2014) | **✓** | **✓** |  | **✓** | **✓** | **✓** | **✓** |  |  |  |  |  |  |  |  |  | **✓** |
| Steiner (2007) |  |  |  | **✓** |  |  |  |  |  |  |  |  |  |  |  |  | **✓** |
| Stevens (2012) |  |  |  |  |  |  |  |  |  |  |  |  |  |  |  |  |  |
| Strele (2012) | **✓** |  |  | **✓** |  |  |  |  |  |  |  |  |  |  |  |  |  |
| Zanders (2013) | **✓** | **✓** |  |  |  |  | **✓** |  | **✓** | **✓** |  |  |  |  |  |  |  |

^a^ Specified comorbidities included one or more of the following: pulmonary dysfunction/disease, prior cardiac event/intervention, vascular disease, hypertension, cardiovascular disease and cerebrovascular disease.

^b^ Specified medications included one or more of the following: contraceptive use, post-menopausal hormone therapy, statin use, metformin use and beta-blocker use.

^c^ Specified reproductive factors included one or more of the following: parity, menopausal status and age at menopause.

^d^ Performance status or American Society of Anaesthesiologists (ASA) score

^e^ Other factors included one or more of the following: alcohol intake, residual disease, tumour diameter, operative complexity, lymphadenectomy, lymph node metastases, period of hormonal activity, income, marital status, geographic region, urban residence, tumour protein p53 status, country, visceral abdominal fat volume, time between diagnosis and interview, study site/group, health care provider, physical activity, waist circumference and invasion of muscle, invasion of endometrium.

^f^ Obesity rather than BMI
