# Supplementary figures and images for: Survival outcomes in endometrial cancer patients according to diabetes: a systematic review and meta-analysis
Source: BMC Cancer. 2022 Apr 20;22:427. doi: 10.1186/s12885-022-09510-7 (PMC9019948; doi:10.1186/s12885-022-09510-7)

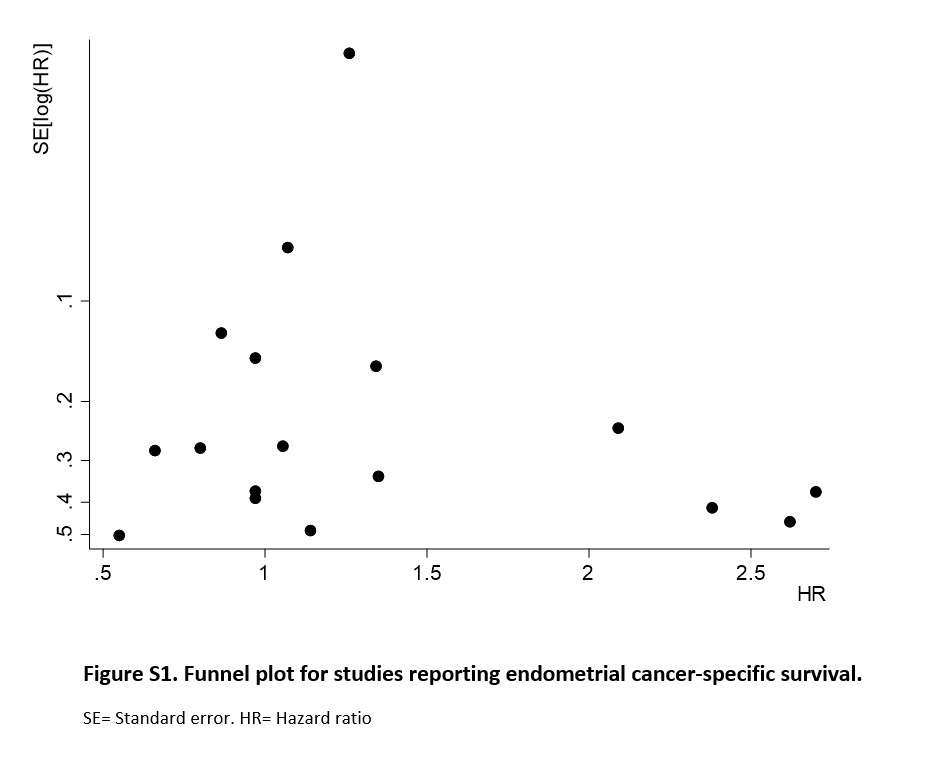

Supplement: Supplementary file 3 — Additional file 3: Figure S1. Funnel plot for cancer-specific survival studies. [file 12885_2022_9510_MOESM3_ESM.jpg]

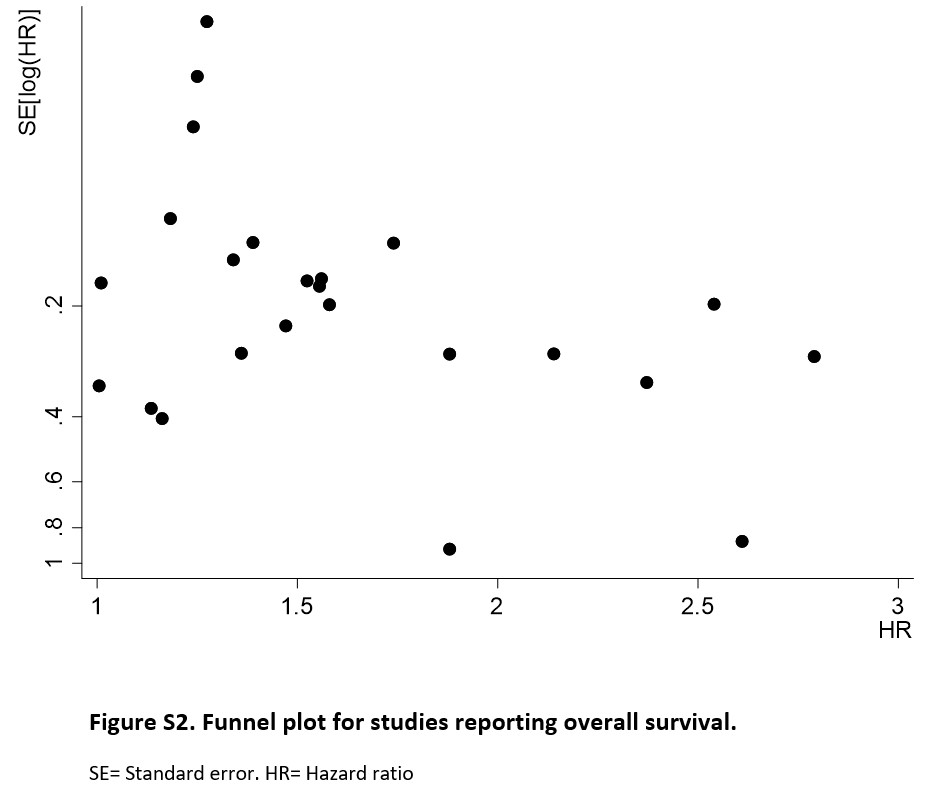

Supplement: Supplementary file 4 — Additional file 4: Figure S2. Funnel plot for overall survival studies. [file 12885_2022_9510_MOESM4_ESM.jpg]

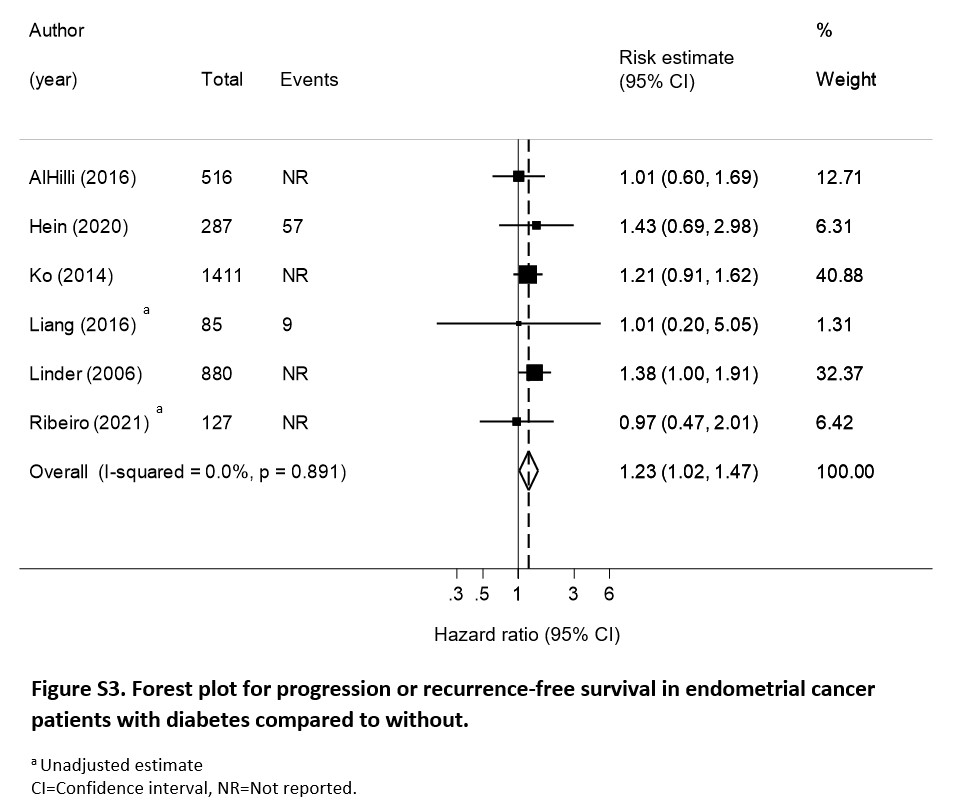

Supplement: Supplementary file 5 — Additional file 5: Figure S3. Forest plot for progression or recurrence-free survival in endometrial cancer patients with diabetes compared to without. [file 12885_2022_9510_MOESM5_ESM.jpg]
