# Supplementary material for: Survival outcomes in endometrial cancer patients according to diabetes: a systematic review and meta-analysis
Source: BMC Cancer. 2022 Apr 20;22:427. doi: 10.1186/s12885-022-09510-7 (PMC9019948; doi:10.1186/s12885-022-09510-7)
Supplement: Supplementary file 6 — Additional file 6: Table S3. Analyses for progression or recurrence-free survival in endometrial cancer patients with diabetes compared to without. [file 12885_2022_9510_MOESM6_ESM.docx]

| **Table S3.**  Summary of main and sub-group analyses for progression/recurrence-free survival in endometrial cancer patients with diabetes compared to those without diabetes. | | | | | |
| --- | --- | --- | --- | --- | --- |
|  | No. of included studies | No. of EC patients | Pooled estimate (95% CI) | I-Squared (%) | P_heterogeneity_ value |
| Main analysis | 6 | 3,306 | 1.23 (1.02-1.47) | 0.0 | 0.89 |
| Multivariate analysis | 4 | 3,094 | 1.25 (1.03-1.51) | 0.0 | 0.75 |
| Univariate analysis | 4 | 2,926 | 1.00 (0.66-1.52) | 56.8 | 0.07 |
| Studies with a quality score of ≤7 | 6 | 3,306 | 1.23 (1.02-1.47) | 0.0 | 0.89 |
| Studies with a follow-up of ≥5 years  **^a^** | 2 | 1,396 | 1.26 (0.96-1.66) | 0.8 | 0.32 |
| Institution-based studies | 6 | 3,306 | 1.23 (1.02-1.47) | 0.0 | 0.89 |
|  | | | | | |
| ^a^ Only included studies which reported a mean or median follow-up.  EC= Endometrial Cancer. CI= Confidence Interval | | | | | |
